# Pneumonia caused by *Pseudomonas fluorescens*: a case report

**DOI:** 10.1186/s12890-021-01573-9

**Published:** 2021-07-05

**Authors:** Xiao Liu, Lei Xiang, Yunhong Yin, Hao Li, Dedong Ma, Yiqing Qu

**Affiliations:** 1 Department of Pulmonary and Critical Care Medicine, Qilu Hospital, Cheeloo College of Medicine, Shandong University, Jinan, 250012 China; 2 Department of Pathology, School of Basic Medical Sciences, Cheeloo College of Medicine, Shandong University, Jinan, 250012 China; 3 Department of Pulmonary and Critical Care Medicine, Qilu Hospital of Shandong University, Wenhuaxi Road 107#, Jinan, 250012 China

**Keywords:** *Pseudomonas fluorescens*, Pneumonia, Fever, Lung biopsy, Case report

## Abstract

**Background:**

*Pseudomonas fluorescens* (*P. fluorescens*) has been detected in respiratory samples from patients. However, no previous reports have been published about these *P. fluorescens* cultures from lung tissues.

**Case presentation:**

Here, we report a case of pneumonia caused by *P. fluorescens*. *P. fluorescens* was identified from lung biopsy specimens for the first time in this case. According to the antibiotic susceptibility testing (AST) of *P. fluorescens*, the patient was given ciprofloxacin treatment. The temperature of the patient then returned to normal. Chest CT examination revealed improvements in pulmonary inflammation.

**Conclusions:**

These findings suggest that the patients with pneumonia caused by *P. fluorescens* should be treated in a timely manner according to the AST results.

## Background

*Pseudomonas fluorescens* (*P. fluorescens*) is a ubiquitous bacterium commonly found in moist environments, such as soil, leaves, and water [1, 2]. As a Gram-negative psychrophile with an optimum growth temperature at 25–30 °C, it is also able to grow at the human body temperature of 37 °C and can present with its virulence factors [3].

*P. fluorescens* is significantly less virulent than *P. aeruginosa* and is a rare cause of invasive hospital-acquired infections, with most common site of infection being the bloodstream [4–15]. It has been isolated in respiratory samples from patients with lung transplants [16–18], ventilator-associated pneumonia (VAP) [19], cystic fibrosis (CF) [20–22] and rice-field drowning-associated pneumonia [23].

While *P. fluorescens* has been identified in human bronchoalveolar lavage fluid (BALF), sputum specimens or throat swabs, its role in pneumonia pathogenesis is unclear. It has been previously suspected of being an aetiologic agent of pneumonia in several reports [19, 24–26], however, the clinical characteristics and drug susceptibility pattern of *P. fluorescens* pneumonia have rarely been reported [25].

In this case, we report a patient with pneumonia caused by *P. fluorescens*. By presenting the clinical and antibiotics susceptibility characteristics of this patient, we will provide significant value for basic and clinical research on *P. fluorescens* infection in the future.

## Case presentation

A 67-year-old man was hospitalized in our hospital complaining of a 10-day history of fever, with a temperature up to 38.8˚C. He denied cough, dyspnoea, chills, shivers or chest pain. Before hospitalization, azithromycin was given orally for 5 days and intravenously for 3 days, and levofloxacin was given intravenously for 1 day. However, the patient still had a fever. He had a past medical history of tuberculosis and gastritis. He was allergic to penicillin, cephalosporin and sulfonamide. He had smoked 10 cigarettes a day for more than 20 years. He retired from an office work and had no pneumotoxic exposure.

**Fig. 1:**
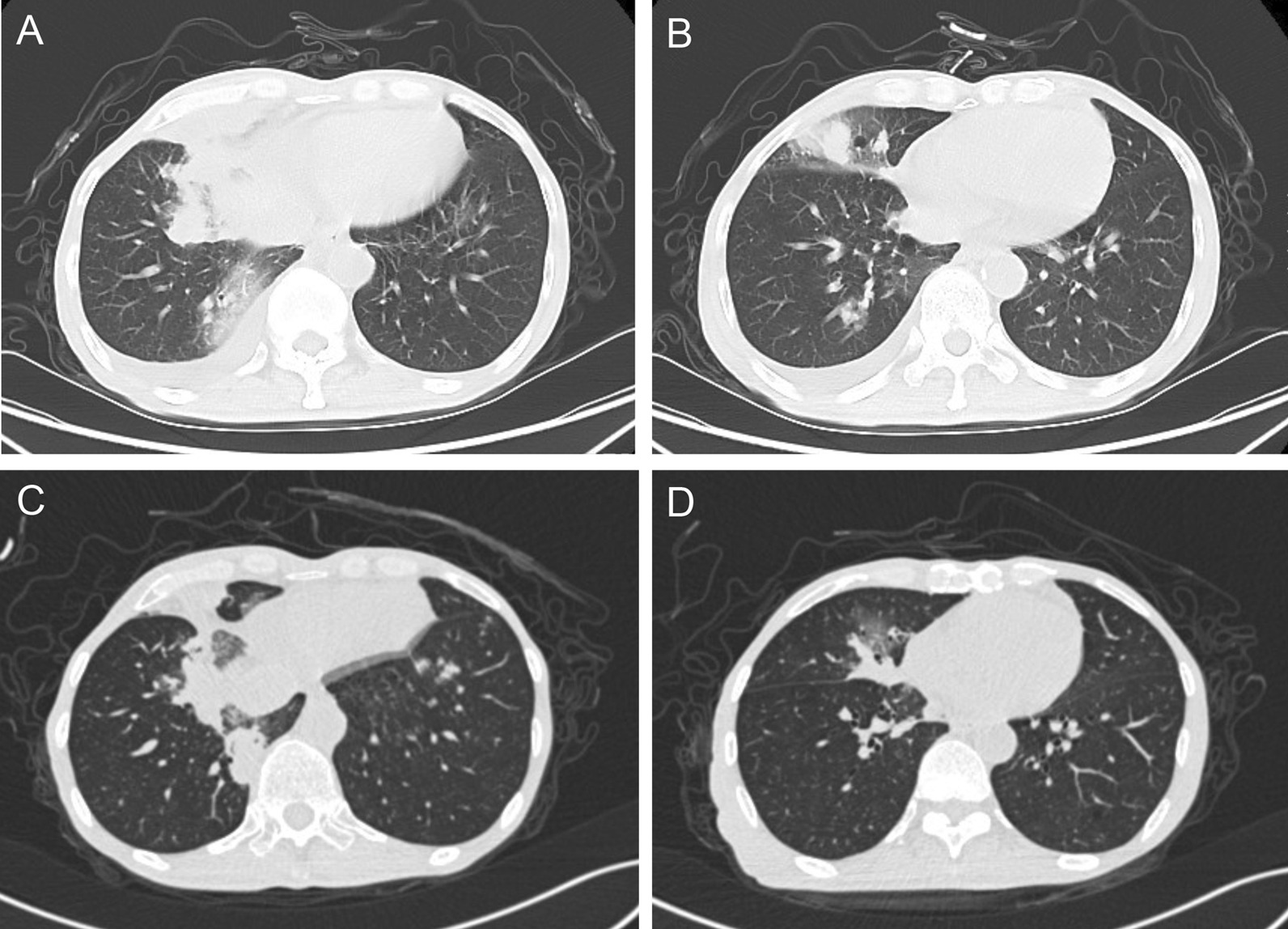
Images of chest computed tomography (CT). **A**, **B** Chest CT images during hospitalization showed high-density infiltrate in bilateral lungs and pleural effusion on the right side. **C**, **D** Chest CT showed the absorption of the pleural effusion and inflammatory sites in the lungs after treatment

At admission, physical examination revealed bilateral reduced breath sounds. The patient was conscious but in a poor state of mind. He was emaciated, with a BMI of 16. The remainder of his physical examination was normal. The results of routine blood examination showed that the white blood cell (WBC) count (10,180/mm^3^ [normal range 3500–95,000/mm^3^]) and neutrophil (NEU) count (8600/mm^3^ [normal range 1800–6300/mm^3^]) were elevated. The erythrocyte sedimentation rate (ESR) (58 mm/h [normal range 0–15 mm/h]) and C-reactive protein (CRP) were increased (96.7 mg/L [normal range 5–10 mg/L]). Albumin was decreased (34.8 g/L [normal range 40–50 g/L]). The chest computed tomography (CT) scan showed scattered patchy high-density nodules with blurred edges in the bilateral lungs. Pleural effusion was present on the right side (Fig. 1A, B).

Anti-mycoplasma pneumonia test and T cell spot test of tuberculosis infection (SPOT. TB) were negative. Thyroid function, rheumatism series, immunoglobulin, complement, respiratory system tumour markers, vasculitis antibodies series, and other laboratory results were normal. The rheumatism series included anti-u1RNP Ab, anti-Smith Ab, anti-SSA Ab, anti-SSB Ab, anti-ScL-70 Ab, anti-PM-SCL Ab, anti-Jo-1 Ab, anti-CENP-B Ab, anti-PCNA Ab, anti-HHT Ab, anti-ZDB Ab, anti-HTT Ab, anti-M2 Ab, ANA, anti-dsDNA Ab, ASL, RF, CRP. The respiratory system tumour markers included CEA, CYFRA21-1, SCC, PRO-GRP, CA-125, NSE. The vasculitis antibodies series included cANCA, PR3-cANCA, pANCA, MPO-pANCA, anti-GBM Ab. In addition, cultures of sputum smears were negative for bacteria, fungi or acid-fast bacilli.

**Fig. 2:**
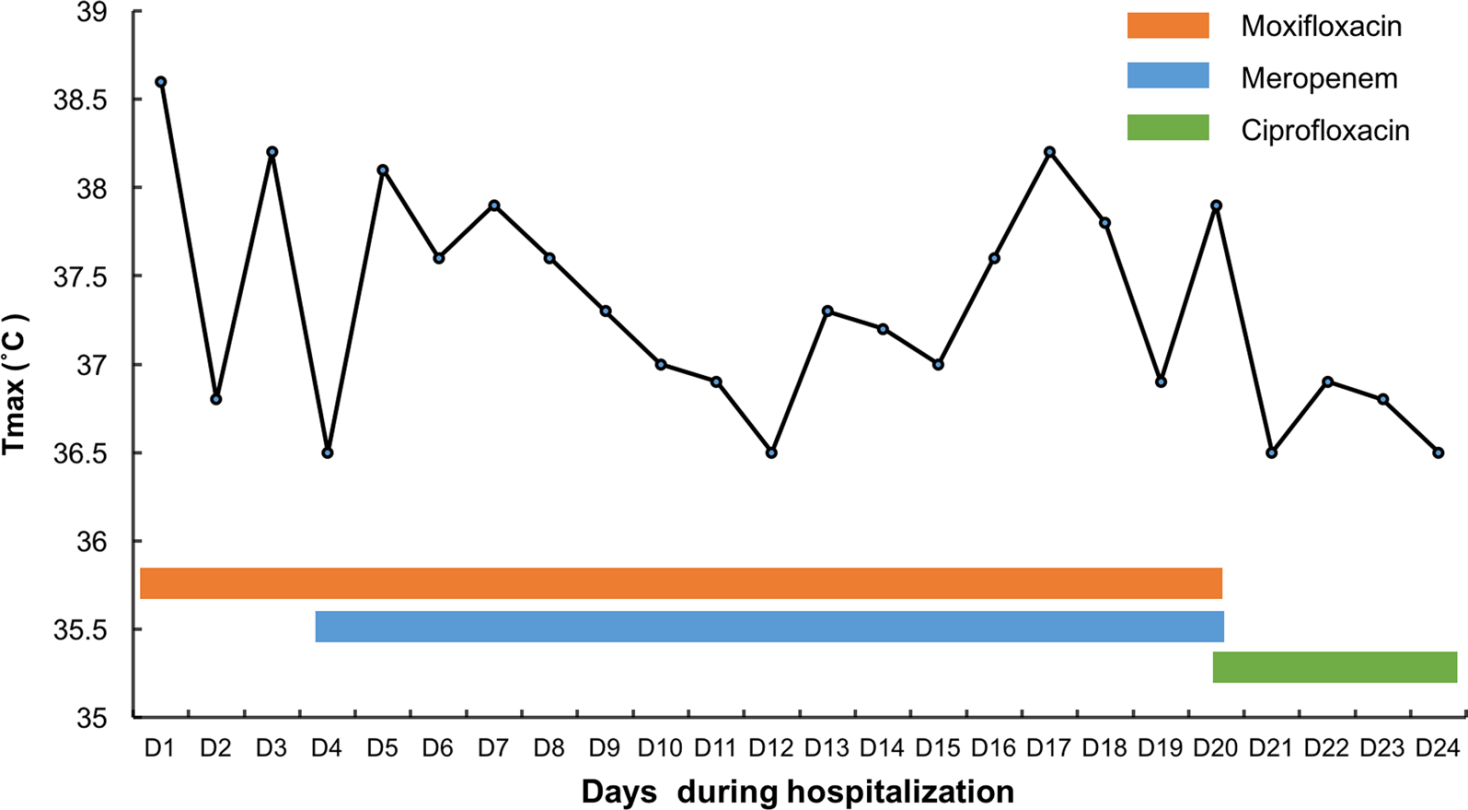
Temperature and medication timeline. Changes in temperature under antibiotic therapy during hospitalization. Tmax, Temperature maximum

After empiric combination treatment with intravenous moxifloxacin (0.4 g, qd) and meropenem (1 g, q8 h) for approximately 2 weeks, the patient still had a fever (Fig. 2). During this period, hydrotalcite (1 g, tid) was given orally, ambroxol (30 mg, bid) and lansoprazole (30 mg, qd) were also given intravenously for dissolving sputum and protecting stomach. Thymopentin was given intramuscularly (20 mg, qd) for improving immunity. On two occasions, dexamethasone (5 mg, st) was given by intravenous injection under a fever in evening. The patient was in poor nutritional status, with difficulties in sputum excretion, making him at high risk for bronchoscopy. Considering these situations, we decided to perform CT-guided lung puncture biopsy for diagnosis.

**Fig. 3:**
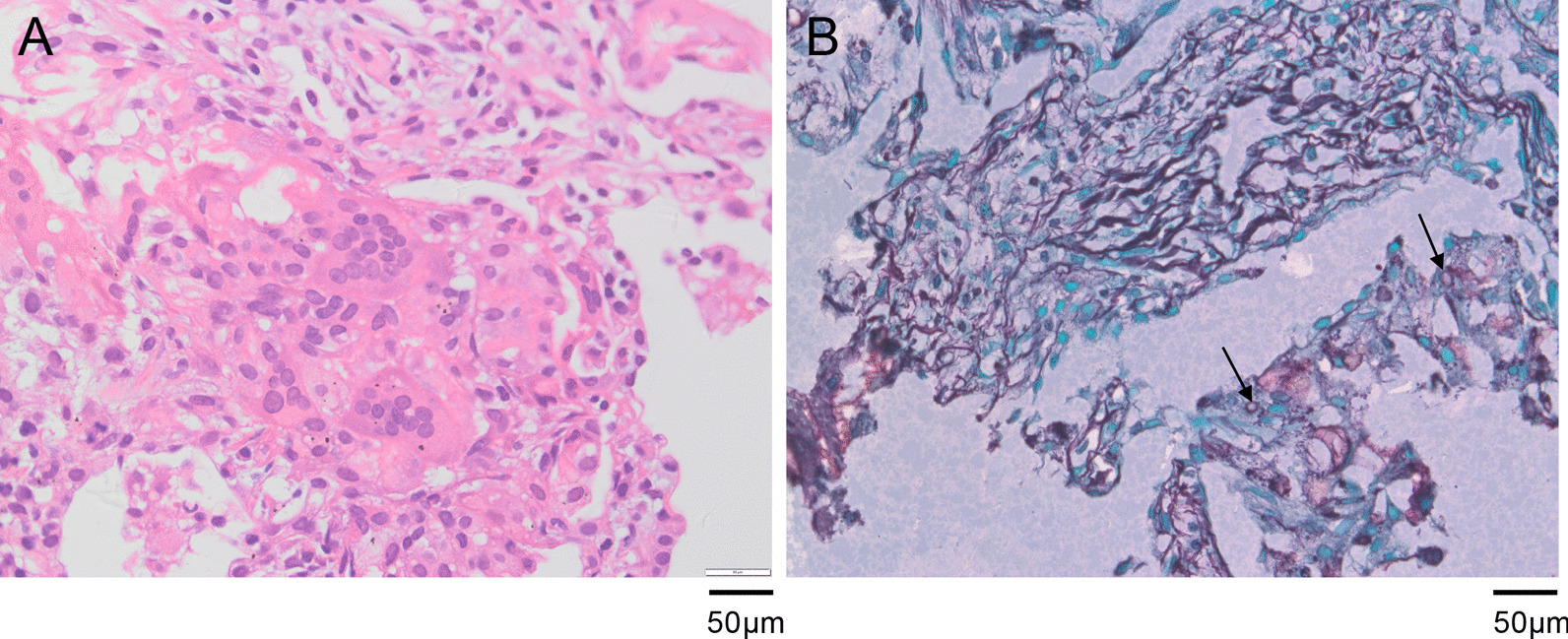
Pathological histology of the biopsy lung tissue. **A** Haematoxylin–eosin staining of lung tissue pathological features showed acute and chronic inflammation, the proliferation of alveolar cells and fibrous tissue, and the existence of multinucleated giant cells. **B** Silver staining of the tissue showed round foreign bodies in foam cells (original magnification, × 400)

The percutaneous lung puncture biopsy was then performed under the guidance of CT. The lung tissue pathological features displayed acute and chronic inflammation, the proliferation of alveolar cells and fibrous tissue, and the existence of multinucleated giant cells (Fig. 3A). Silver staining of the tissue showed round foreign bodies in foam cells (Fig. 3B). The identification of the organism types in lung tissue was performed by standard biochemical tests using a standard method on a Vitek 2-GN ID card (bioMerieux, Marcy l'Etoile, France). *P. fluorescens* was identified, and Vitek 2 antibiotic susceptibility testing (AST) of *P. fluorescens* was performed. The results of AST showed that *P. fluorescens* was susceptible to ceftazidime, ciprofloxacin, cefepime, amikacin, gentamicin, tobramycin, piperacillin-tazobactam, and levofloxacin. Meanwhile, the *P. fluorescens* was resistant to ampicillin, cefazolin, imipenem, sulfamethoxazole/trimethoprim, ampicillin/sulbactam, cefotetan, and ceftriaxone (Table 1). According to the susceptibility pattern, moxifloxacin and meropenem were discontinued, and ciprofloxacin (0.4 g, bid) was administered for 4 days. The patient had no fever during treatment with ciprofloxacin (Fig. 2). Then, the patient was discharged and continued to use oral ciprofloxacin.

**Table 1:** The antimicrobial susceptibility testing results of *P. fluorescens*

Antibiotics	MIC values (μg/ml)	Interpretation
Ampicillin	≥ 32	R
Cefazolin	≥ 64	R
Ceftazidime	8	S
Ciprofloxacin	≤ 0.25	S
Imipenem	≥ 16	R
Sulfamethoxazole/trimethoprim	80	R
Cefepime	4	S
Amikacin	≤ 2	S
Ampicillin/sulbactam	≥ 32	R
Cefotetan	≥ 64	R
Ceftriaxone	≥ 64	R
Gentamicin	≤ 1	S
Tobramycin	≤ 1	S
Piperacillin-tazobactam	≤ 4	S
Levofloxacin	0.5	S

Susceptibility cards were inoculated and interpreted according to the Clinical and Laboratory Standards Institute (CLSI) breakpoints.

*P. fluorescens* *Pseudomonas fluorescens*, *MIC* minimum inhibitory concentration, *R*  resistant, *S* sensitive.

The patient had a return visit one month later. He denied clinical symptoms. The WBC and NEU counts of his blood samples were in the normal range. Chest CT showed the absorption of the pleural effusion and the inflammatory sites in the lungs (Fig. 1C, D). Six months later, the patient returned again and bronchoscopy was performed. No bacteria, acid-fast bacilli, fungi or spores was found in the bronchial brushings and BALF specimen.

## Discussion and conclusion

Here, we report a case of pneumonia caused by *P. fluorescens*. *P. fluorescens* was cultured from the biopsy lung tissue of this patient. Based on the AST results of *P. fluorescens*, the condition of this patient improved in response to ciprofloxacin therapy. In previous studies of *P. fluorescens*, the clinical samples included sputum, BALF or throat swabs. In contrast to previous reports, we report *P. fluorescens* cultured from lung biopsy specimens for the first time.

The roles of *P. fluorescens* in pneumonia or other respiratory diseases pathogenesis are undefined. The clinical features of *P. fluorescens*-associated pneumonia have rarely been reported [25]. In the case of a myasthenic patient [25], during recovery from a recent polymicrobial peritonitis, he developed clinical evidence of pneumonia, with sputum cultures that were positive for *P. fluorescens*. Prior to the pneumonia, this 55-year-old patient received the treatment with intravenous cefuroxime and metronidazole, and mechanical ventilation. The *P. fluorescens* was sensitive to amikacin, gentamicin, tobramycin, netilmicin, piperacillin, ticarcillin, latamoxef and ceftazidime. He recovered after the therapy of metronidazole supplemented with intravenous ceftazidime. *P. fluorescens* was also mentioned in the aetiology of community-acquired pneumonia in a single case, and the *P. fluorescens* did not respond to therapy with ceftriaxone, but the clinical details were lacking [26].

In most previous studies, it is unclear whether the positive sputum or BALF culture results reflected acute infection or benign colonization of patients. Using amplification of bacterial 16S rRNA genes, *P. fluorescens* and other bacteria was detected in the BALF acquired from a single patient with VAP [19]. In another study of over 1,000 respiratory cultures acquired from patients with CF, the authors identified *P. fluorescens* in approximately 2% of samples and considered the organism a colonizer rather than an acute pathogen [22]. *P. fluorescens* was also identified in a single patient with CF and new lower airways infection [20]*.* In a survey of lung transplant recipients, *P. fluorescens* was frequently identified in BALF samples of asymptomatic recipients by pyrosequencing, but not detected via standard bacterial culture [18].

In their survey [18], researchers also searched the database of bacterial culture isolates from the University of Michigan Clinical Microbiology Laboratory. Over an 11-year period, *P. fluorescens* was cultured from over 240 distinct respiratory specimens, including sputum, throat swabs, and bronchoscopically-obtained specimens (BALF or brushings). Among patients with positive *P. fluorescens* respiratory cultures, the most common underlying pulmonary condition was CF (38.8% of all isolates), followed by other chronic airway diseases (COPD, asthma, and non-CF bronchiectasis [16.1%]) and lung transplantation (7.4%). In addition, 26 of these specimens were obtained from patients with suspected acute pneumonia, and 22 of these patients were chronically immunosuppressed or had recent healthcare exposures meeting criteria for healthcare-associated pneumonia.

In our report, the patient had none known risk factor for *P. fluorescens* colonization or infection, including ventilator usage, lung transplantation, CF, immunosuppression, or other chronic airway diseases. However, it is worth noting that this eldly patient was thin and had low albumin level, with a smoking history of more than 20 years. And he received the treatment with multiple antibiotics before lung biopsy.

The patient presented with a fever and radiographic lung infiltrate. Laboratory examinations revealed elevated WBC and NEU counts. He was in poor nutritional status, with difficulties in sputum excretion, making him at high risk for bronchoscopy. Therefore, the CT-guided lung puncture biopsy was performed for diagnosis. *P. fluorescens* was cultured from lung biopsy specimens. The clinical symptoms, CT and laboratory test results, pathologic findings, and treatment response to ciprofloxacin provide evidence of *P. fluorescens* infection in our case. The pathohistological diagnosis of the biopsy provided meaning guidance for a clinical diagnosis, including the exclusion of tumours, granulomatous diseases, TB infection, fungal infection, etc. However, pathological observation cannot identify the type of bacterial pathogens. More importantly, the results of tissue culture and drug sensitivity tests played an important role in guiding the use of antibiotics to treat this patient.

In the AST, *P. fluorescens* was resistant to multiple antibiotics, which may be the reason for the poor efficacy of initial empirical therapy. Notably, the antimicrobial susceptibility results of our *P. fluorescens* isolates were in agreement with known findings as described above [25, 26]. They were resistant to ceftriaxone, and sensitive to amikacin, gentamicin, tobramycin, ceftazidime and piperacillin. However, the previous studies relating antimicrobial susceptibility characteristics were early and few, the coverage of antibiotics was relatively narrow. The antimicrobial susceptibility patterns of *P. fluorescens* pneumonia need to be further summarized and clarified.

In summary, *P. fluorescens* can cause acute pneumonia, with fever as the main clinical symptom. When encountering patients with pneumonia presenting with poor efficacy of empiric antibiotic treatment, we should consider the possibility of *P. fluorescens* infection. In addition, it is important to perform the bacterial culture and AST in a timely manner. Antibiotic therapy under the guidance of the *P. fluorescens* antimicrobial spectrum is significant for such patients. However, more research is needed to study the pathogenesis of *P. fluorescens* and to establish diagnostic criteria and effective treatment of these cases.

## Data Availability

Not applicable.

## Abbreviations

*P. fluorescens*: *Pseudomonas fluorescens*
CF: Cystic fibrosis
VAP: Ventilator-associated pneumonia
BALF: Bronchoalveolar lavage fluid
WBC: White blood cell
NEU: Neutrophil
ESR: Erythrocyte sedimentation rate
CRP: C-reactive protein
SPOT.TB: T cell spot test of tuberculosis infection
CT: Chest computed tomography
Ab: Antibody
u1RNP: U1-ribonucleoprotein
SSA: Sjögren’s syndrome A
SSB: Sjögren’s syndrome B
ScL-70: Topoisomerase I
PM-SCL: Polymyositis-sclerosis
Jo-1: Cytoplasmic aminoacyl-tRNA synthetase
CENP-B: Centromere protein B
PCNA: Proliferating cell nuclear antigen
HHT: Nucleosome
ZDB: Histone
HTT: Ribosomal P protein
M2: Mitochondrial antibody subtype M2
ANA: Anti-nuclear Ab
dsDNA: Double-stranded DNA
ASL: Anti-streptolysin O
RF: Rheumatoid factors
CRP: C-reactive protein
CEA: Carcinoembryonic antigen
CYFRA21-1: Cytokeratin 19 fragment antigen 21-1
SCC: Squamous cell carcinoma antigen
PRO-GRP: Pro-gastrin releasing peptide
CA-125: Carbohydrate antigen 125
NSE: Neuron-specific enolase
cANCA: Cytoplasmic anti-neutrophil cytoplasmic antibody
PR3: Proteinase-3
pANCA: Perinuclear anti-neutrophil cytoplasmic antibody
MPO: Myeloperoxidase
GBM: Glomerular basement membrane
COPD: Chronic obstructive pulmonary disease
